# Data for developing allometric models and evaluating carbon stocks of the Zambezi Teak Forests in Zambia

**DOI:** 10.1016/j.dib.2018.02.057

**Published:** 2018-02-28

**Authors:** Justine Ngoma, Eddy Moors, Bart Kruijt, James H. Speer, Royd Vinya, Emmanuel N. Chidumayo, Rik Leemans

**Affiliations:** a School of Natural Resources, The Copperbelt University, P.O. Box 21692, Kitwe, Zambia; b Water Systems and Global Change Group, Wageningen University and Research, P.O Box 47, 6700AA Wageningen, The Netherlands; c Department of Earth and Environmental Systems, Indiana State University, Terre Haute, IN 47809, USA; d VU University Amsterdam, De Boelelaan 1085, 1081 HV Amsterdam, The Netherlands; e Environmental Systems Analysis Group, Wageningen University and Research, P.O Box 47, 6700AA Wageningen, The Netherlands; f Makeni Savanna Research Project, P.O Box 50323, Ridgeway, Lusaka, Zambia; g IHE Delft Institute for Water Education, PO Box 3015, 2601 DA Delft, The Netherlands

## Abstract

This paper presents data on carbon stocks of tropical tree species along a rainfall gradient. The data was generated from the Sesheke, Namwala, and Kabompo sites in Zambia. Though above-ground data was generated for all these three sites, we uprooted trees to determine below-ground biomass from the Sesheke site only. The vegetation was assessed in all three sites. The data includes tree diameter at breast height (DBH), total tree height, wood density, wood dry weight and root dry weight for large (≥ 5 cm DBH) and small (< 5 cm DBH) trees. We further presented Root-to-Shoot Ratios of uprooted trees. Data on the importance-value indices of various species for large and small trees are also determined. Below and above-ground carbon stocks of the surveyed tree species are presented per site. This data were used by Ngoma et al. (2018) [1] to develop above and below-ground biomass models and the reader is referred to this study for additional information, interpretation, and reflection on applying this data.

**Specifications Table**

**Table:** 

**Subject area**	**Ecology**
More specific subject area	Carbon stocks of the Zambezi Teak Forests.
Type of data	Tables and Figures.
How data was acquired	We generated data to develop above-ground and below-ground biomass models by respectively cutting down trees and uprooting trees. We assessed vegetation characteristics by generating data to determine carbon stocks. We determined the carbon fractions in leaves, branches, stems, and roots from all cut and uprooted trees. These carbon fractions were measured in the laboratory using a Fisons EA1108 CHN-0 elemental analyser (See Ngoma et al. [1] for details).
Data format	Analyzed and Raw.
Experimental factors	Root and wood samples were immediately weighed whilst in the field. Samples taken to develop allometric models were then oven dried for 24 h at 105 °C to obtain their dry weight after determining their volume through the water-displacement approach in the ‘as received condition’ [1], [2]. Stem, branches, roots, and leaves were ground into fine powder before analyzing them for their C fraction. Wood volume was not measured for the disk samples that were taken to determine their carbon fraction.
Experimental features	Data were collected along a rainfall gradient covering high, intermediate and low rainfall areas (See Ngoma et al. [1] for details).
Data source location	We collected data from Kabompo (14°00.551S, 023°35.106E), Namwala (15°50.732S, 026°28.927E), and Sesheke (17°21.278S, 24°22.560E) in Zambia.
Data accessibility	Data are provided in this paper to improve data accessibility and further data at the tree-level are available online in excel format (Supplementary information 1 and 2.

**Value of the data**

• The data can be used to understand carbon-stock distributions for a tropical precipitation gradient. This gives insights on how climate change likely affects these distributions.
• The data provide information on the carbon-storage potential of various species, thereby giving insight on the carbon-sequestration potential of individual species.
• The average root-to-shoot ratio presented can be applied in similar forests to determine the below-ground biomass stocks from the above-ground biomass values; and
• The data can be used to develop allometric models of similar tropical forests types and species.

## 1 Data

We present data on various tree parameters (e.g. diameter at breast height (DBH), total tree height, wood density, and dry weight). The data presented in Section 1.1 were used to determine carbon fraction in leaves, stem, branches, and roots, and to develop above and below-ground biomass models. Root-to-Shoot ratios of the uprooted trees were also calculated. Section 1.2 provides the species-importance-value (SIV) indices of all surveyed trees, which are categorized as large (≥ 5 cm DBH) or small (< 5 cm DBH) trees. In Section 1.3, data on carbon stocks of various surveyed tree species per study site are presented.

### 1.1 Parameters of trees used to develop allometric models

See Table 1, Table 2, Table 3 here.

**Table 1:** Diameter (DBH), total tree height, wood density, and wood dry weight of sampled small trees (< 5 cm DBH).

**Species**	**Diameter (DBH, cm)**	**Total tree height (m)**	**Wood density (g/m**^**3**^**)**	**Wood dry weight (kg)**
*Baphia massaiensis*	2.30	3.00	0.77	0.66
*Baphia massaiensis*	1.10	2.00	0.56	0.24
*Baphia massaiensis*	3.50	3.95	0.66	2.10
*Baphia massaiensis*	2.50	2.35	0.67	0.69
*Baphia massaiensis*	1.80	3.20	0.70	0.72
*Baphia massaiensis*	3.50	4.70	0.70	3.04
*Baphia massaiensis*	4.50	7.00	0.73	5.58
*Baphia massaiensis*	4.50	4.90	0.70	4.10
*Baphia massaiensis*	1.10	2.50	0.79	3.32
*Baphia massaiensis*	1.20	3.40	0.93	3.01
*Baphia massaiensis*	2.50	4.40	0.95	0.91
*Baphia massaiensis*	3.00	4.90	0.92	0.36
*Baphia massaiensis*	3.70	5.50	0.88	1.75
*Baphia massaiensis*	3.40	5.30	0.76	2.12
*Baphia massaiensis*	4.60	4.00	0.89	2.07
*Baphia massaiensis*	4.50	4.50	0.94	0.60
*Baphia massaiensis*	1.80	3.70	0.73	0.86
*Baphia massaiensis*	2.40	3.80	1.07	0.33
*Baphia massaiensis*	1.50	3.70	1.04	0.23
*Baphia massaiensis*	3.10	4.90	0.72	4.79
*Baphia massaiensis*	2.80	4.00	0.62	0.28
*Baphia massaiensis*	1.50	3.90	0.86	0.35
*Baphia massaiensis*	2.00	3.50	0.88	0.40
*Baphia massaiensis*	1.60	3.58	0.73	0.26
*Baphia massaiensis*	2.40	3.80	0.75	0.14
*Baphia massaiensis*	1.60	3.20	1.01	0.35
*Baphia massaiensis*	1.10	2.50	0.71	0.17
*Combretum celastroides*	2.20	2.10	0.08	2.07
*Combretum celastroides*	2.00	3.85	0.93	1.66
*Combretum celastroides*	1.30	3.10	0.99	2.49
*Combretum celastroides*	3.10	4.40	0.93	3.60
*Combretum celastroides*	4.50	5.00	0.97	2.82
*Combretum celastroides*	4.20	4.70	0.90	0.97
*Combretum celastroides*	2.80	3.30	0.83	2.04
*Combretum celastroides*	3.80	8.40	0.97	3.82
*Diplorhynchus candylocarpon*	4.50	4.20	0.53	0.91
*Diplorhynchus candylocarpon*	4.70	5.30	0.46	0.33
*Diplorhynchus candylocarpon*	2.80	8.90	0.47	2.04
*Diplorhynchus candylocarpon*	1.40	4.15	0.65	7.22
*Diplorhynchus candylocarpon*	3.40	4.30	0.76	6.16
*Diplorhynchus candylocarpon*	3.50	5.70	0.69	0.42
*Diplorhynchus candylocarpon*	1.60	3.40	0.70	0.58
*Diplorhynchus candylocarpon*	2.80	5.40	0.54	3.71
*Friesodielsia obovata*	1.60	3.10	0.51	0.38
*Friesodielsia obovata*	1.20	2.80	0.84	9.57
*Friesodielsia obovata*	4.80	8.80	0.70	2.32
*Friesodielsia obovata*	3.20	4.00	0.81	0.64
*Friesodielsia obovata*	2.70	4.00	0.72	0.72
*Friesodielsia obovata*	2.70	3.00	0.85	2.78
*Friesodielsia obovata*	4.00	3.60	0.78	0.25
*Friesodielsia obovata*	1.20	3.40	0.70	0.55
*Friesodielsia obovata*	2.40	4.70	0.68	0.39
*Friesodielsia obovata*	2.60	5.01	0.63	1.03
*Friesodielsia obovata*	3.10	5.05	0.72	1.30
*Friesodielsia obovata*	3.10	4.10	0.67	5.63
*Friesodielsia obovata*	1.60	4.04	0.89	0.26
*Friesodielsia obovata*	4.90	6.90	0.75	3.01
*Pteleopsis anisoptera*	3.20	6.10	0.68	6.28
*Pteleopsis anisoptera*	2.60	5.20	0.72	0.35
*Pteleopsis anisoptera*	2.40	2.90	0.68	4.35
*Pteleopsis anisoptera*	1.20	3.20	0.73	0.48
*Pteleopsis anisoptera*	1.10	3.40	0.87	0.37
*Pteleopsis anisoptera*	4.90	5.60	0.75	3.03
*Pteleopsis anisoptera*	3.10	5.80	0.70	1.92
*Pteleopsis anisoptera*	4.70	6.50	0.81	3.70
*Pterocarpus antunesii*	4.30	8.00	0.79	0.40
*Pterocarpus antunesii*	4.40	7.00	0.89	0.88
*Pterocarpus antunesii*	1.40	4.10	0.50	0.39
*Pterocarpus antunesii*	3.80	5.80	0.80	1.14
*Pterocarpus antunesii*	2.30	5.40	1.19	0.49
*Pterocarpus antunesii*	1.70	5.20	0.79	0.32
*Pterocarpus antunesii*	4.00	7.40	0.69	0.77
*Pterocarpus antunesii*	3.80	7.25	0.78	4.54
*Pterocarpus antunesii*	4.30	5.30	0.73	5.97

**Table 2:** Diameter (DBH), total tree height, wood density, and wood dry weight of sampled large trees (≥ 5 cm DBH).

**Species**	**Diameter (DBH, cm)**	**Total height (m)**	**Wood density (g/m**^**3**^**)**	**Wood dry weight (kg)**
*Baikiaea plurijuga*	32.50	12.44	0.83	459.99
*Baikiaea plurijuga*	34.00	15.32	0.96	619.83
*Baikiaea plurijuga*	21.00	11.95	0.78	129.50
*Baikiaea plurijuga*	7.00	8.20	0.92	14.80
*Baikiaea plurijuga*	26.70	14.90	1.00	271.89
*Baikiaea plurijuga*	33.00	9.80	0.94	493.30
*Baikiaea plurijuga*	48.70	17.55	0.88	1031.10
*Baikiaea plurijuga*	43.70	16.90	0.91	944.59
*Baikiaea plurijuga*	55.50	16.90	0.94	2043.49
*Baikiaea plurijuga*	51.00	17.85	0.89	1020.93
*Baikiaea plurijuga*	69.50	21.90	0.91	2355.53
*Baikiaea plurijuga*	39.50	39.50	0.85	949.69
*Baikiaea plurijuga*	22.20	11.95	0.80	204.77
*Baikiaea plurijuga*	33.10	14.00	0.85	423.57
*Baikiaea plurijuga*	41.00	15.19	1.01	744.65
*Baikiaea plurijuga*	43.00	14.20	0.91	689.72
*Baikiaea plurijuga*	8.50	6.70	0.92	13.30
*Baikiaea plurijuga*	12.00	9.80	0.94	55.06
*Baikiaea plurijuga*	12.00	10.05	0.85	54.89
*Baikiaea plurijuga*	8.00	7.65	0.69	17.60
*Baikiaea plurijuga*	50.00	15.37	0.83	1321.92
*Baikiaea plurijuga*	25.00	10.80	0.98	310.46
*Baikiaea plurijuga*	44.00	14.90	0.90	1201.18
*Baikiaea plurijuga*	35.00	6.20	1.02	427.83
*Baikiaea plurijuga*	21.20	10.30	0.75	181.39
*Baikiaea plurijuga*	25.00	11.37	0.89	307.05
*Baikiaea plurijuga*	26.00	12.40	1.15	489.81
*Baikiaea plurijuga*	41.00	12.50	0.85	947.46
*Baikiaea plurijuga*	29.00	10.30	0.73	169.89
*Baikiaea plurijuga*	13.70	13.20	0.78	63.18
*Baikiaea plurijuga*	42.20	12.12	0.88	892.87
*Baikiaea plurijuga*	33.00	10.17	1.15	917.50
*Baikiaea plurijuga*	23.70	12.10	0.83	232.91
*Baikiaea plurijuga*	51.50	11.80	0.93	1294.72
*Baikiaea plurijuga*	16.50	10.40	0.94	29.27
*Baikiaea plurijuga*	46.30	10.42	0.97	961.39
*Baikiaea plurijuga*	62.00	19.30	0.86	2659.55
*Baphia massaiensis*	10.00	7.85	0.77	17.80
*Baphia massaiensis*	16.00	9.05	0.89	64.46
*Baphia massaiensis*	13.00	9.25	0.78	65.57
*Baphia massaiensis*	35.00	12.70	0.86	467.63
*Baphia massaiensis*	20.00	8.47	0.84	63.11
*Baphia massaiensis*	16.00	7.80	1.02	38.65
*Baphia massaiensis*	7.50	8.60	0.98	16.79
*Combretum hereroense*	24.00	18.40	0.88	201.16
*Combretum hereroense*	25.00	6.62	0.67	212.95
*Combretum hereroense*	41.50	12.22	0.79	805.12
*Combretum hereroense*	11.00	34.50	0.65	25.63
*Combretum hereroense*	16.00	9.08	0.70	36.96
*Combretum hereroense*	5.00	6.60	0.62	6.52
*Combretum hereroense*	36.50	13.50	0.81	361.30
*Combretum hereroense*	9.00	20.90	0.62	20.09
*Combretum hereroense*	38.40	15.35	0.88	556.10
*Diplorhynchus candylocarpon*	10.00	6.30	0.67	27.49
*Diplorhynchus candylocarpon*	14.40	6.50	0.75	61.05
*Diplorhynchus candylocarpon*	28.50	8.55	0.92	161.07
*Diplorhynchus candylocarpon*	22.00	7.56	0.72	117.92
*Diplorhynchus candylocarpon*	33.00	7.60	0.73	274.79
*Diplorhynchus candylocarpon*	15.00	5.65	0.83	38.07
*Diplorhynchus candylocarpon*	9.70	4.85	0.80	19.32
*Diplorhynchus candylocarpon*	22.00	8.90	0.76	296.68
*Diplorhynchus candylocarpon*	5.10	4.25	0.48	5.53
*Diplorhynchus candylocarpon*	5.50	4.70	0.63	3.27
*Entandrophragma caudatum*	36.00	17.30	0.64	563.02
*Entandrophragma caudatum*	46.50	16.07	0.65	193.80
*Ficus sycomorus*	17.00	7.75	0.70	95.26
*Ficus sycomorus*	15.70	6.70	0.78	76.48
*Ficus sycomorus*	23.00	5.65	0.56	193.80
*Ficus sycomorus*	16.50	7.48	0.99	103.44
*Ficus sycomorus*	17.00	5.56	0.68	56.30
*Lonchocarpus nelsii*	9.50	6.40	0.99	19.54
*Lonchocarpus nelsii*	29.00	11.30	1.11	300.64
*Lonchocarpus nelsii*	16.00	8.75	0.80	75.21
*Lonchocarpus nelsii*	16.20	6.60	0.69	59.37
*Pteleopsis anisoptera*	5.00	7.50	0.99	6.57
*Pteleopsis anisoptera*	10.00	9.00	0.86	37.04
*Pteleopsis anisoptera*	9.00	9.20	0.83	16.57
*Pteleopsis anisoptera*	15.20	11.30	0.66	57.46
*Pteleopsis anisoptera*	27.00	14.75	0.95	315.11
*Pteleopsis anisoptera*	28.00	16.45	0.97	543.42
*Pteleopsis anisoptera*	31.70	13.60	0.98	365.56
*Pteleopsis anisoptera*	34.00	16.63	0.85	590.86
*Pteleopsis anisoptera*	33.00	18.30	1.13	422.99
*Pterocarpus angolensis*	19.00	8.09	0.56	66.13
*Pterocarpus angolensis*	6.30	4.85	0.42	6.16
*Pterocarpus angolensis*	10.00	5.85	0.68	27.08
*Pterocarpus angolensis*	13.50	7.55	0.47	39.09
*Pterocarpus angolensis*	24.00	10.15	0.59	159.04
*Pterocarpus angolensis*	21.60	10.80	0.64	169.62
*Pterocarpus angolensis*	31.50	9.48	0.56	365.62
*Pterocarpus angolensis*	12.00	6.19	0.76	43.72
*Pterocarpus angolensis*	50.30	11.88	0.70	1488.17
*Pterocarpus angolensis*	32.50	11.75	0.62	199.30
*Pterocarpus angolensis*	43.00	14.44	0.65	803.22
*Pterocarpus antunesii*	39.00	14.05	0.95	895.36
*Pterocarpus antunesii*	19.00	16.55	0.72	182.70
*Pterocarpus antunesii*	20.00	18.55	0.93	160.68
*Pterocarpus antunesii*	10.00	11.50	0.76	28.62
*Pterocarpus antunesii*	6.50	10.40	0.67	9.50
*Pterocarpus antunesii*	32.00	11.87	0.69	630.64
*Pterocarpus antunesii*	23.00	12.23	0.80	401.37
*Pterocarpus antunesii*	44.00	18.81	0.83	651.66
*Pterocarpus antunesii*	41.00	15.26	0.83	1170.64
*Pterocarpus antunesii*	25.00	13.60	0.73	205.56

**Table 3:** Diameter (DBH), total tree height, wood density, wood dry weight, and root dry weight of sampled uprooted trees.

**Species**	**DBH (cm)**	**Total tree height (m)**	**Above-ground biomass (Kg)**	**Root density (g/m**^**3**^**)**	**Root biomass (Kg)**	**Root-to-Shoot ratio**
*Baikiaea plurijuga*	25	11	310	0.89	56	0.18
*Baikiaea plurijuga*	44	18	1201	0.85	295	0.25
*Baikiaea plurijuga*	35	6	428	0.67	151	0.35
*Ficus sycomorus*	17	8	95	0.53	27	0.28
*Lonchocarpus nelsii*	10	6	20	0.76	9	0.47
*Lonchocarpus nelsii*	29	11	301	0.80	199	0.66
*Ficus sycomorus*	16	7	76	0.48	35	0.46
Average						**0.38**

### 1.2 Species importance value indices of large (≥ 5 cm DBH) and small (< 5 cm DBH) trees

This section provides the SIV indices of all surveyed trees and tree species [1] (see Table 4, Table 5). Indices were calculated following the Cottam and Curtis [3] method. Supplementary information 1 (small trees) and 2 (large trees) provide a list of all trees and tree species surveyed. The information are excel files and available in electronic format.

**Table 4:** Species Importance Value (SIV) Indices of small trees (< 5 cm DBH) per site. (Note: A dash means that a species was not found at the site).

**Species**	**Kabompo**	**Namwala**	**Sesheke**	**Language of the species name**
*Acacia ataxacantha*	–	–	11.01	Botanical
*Afzelia quanzensis*	3.07	–	–	Botanical
*Baikiaea plurijuga*	–	4.82	–	Botanical
*Baphia massaiensis*	70.37	69.68	27.08	Botanical
*Bauhinia petersiana*	–	–	0.00	Botanical
*Brachystegia speciformis*	8.27	–	–	Botanical
*Cassia abbreviata*	–	4.90	–	Botanical
*Combretum celastroides*	–	30.96	0.00	Botanical
*Combretum hereroense*	–	–	15.51	Botanical
*Combretum molle*	–	9.07	–	Botanical
*Combretum zeyheri*	–	1.37	–	Botanical
*Commiphora mollis*	–	3.57	–	Botanical
*Croton gratissimus*	–	–	0.00	Botanical
*Dichrostachys cinerea*	–	1.35	–	Botanical
*Diplorhynchus candylocarpon*	49.44	26.78	4.82	Botanical
*Eucalyptus (exotic species)*	–	–	0.00	Botanical
*Friesodielsia obovata*	3.01	45.03	42.71	Botanical
*Hippocratea africana*	–	–	5.09	Botanical
*Hymenocardia acida*	–	1.37	–	Botanical
*Ibu*	–	1.32	–	Ila
*Kapasa ka lyongono*	1.54	–	–	Luvale
*Lonchocarpus nelsii*	–	–	12.23	Botanical
*Mang'omba*	–	3.19	–	Tonga
*Markhamia obtusifolia*	2.06	17.95	–	Botanical
*Markhamia zanzibarica*	–	–	12.47	Botanical
*Mbungeimo*	–	–	0.00	Lozi
*Mubangabanga*	6.64	–	–	Luvale
*Mubeba*	1.65	–	–	Luvale
*Mubwabwa*	–	2.77	–	Luvale
*Muhoho*	1.35	–	–	Luvale
*Muhuhu*	12.67	–	–	Luvale
*Mukube*	5.56	–	–	Luvale
*Mumbukushu*	10.84	–	–	Luvale
*Mumbumelenge*	–	–	0.00	Lozi
*Mutungambabala*	–	1.70	–	Tonga
*Mwingili*	–	1.34	–	Tonga
*Namulomo*	–	–	25.73	Lozi
*Pseudolachnostylis maprouneifolia*	–	3.22	43.67	Botanical
*Pteleopsis anisoptera*	22.28	–	–	Botanical
*Pterocarpus angolensis*	–	1.34	–	Botanical
*Pterocarpus angolensis*	–	40.17	–	Botanical
*Pterocarpus antunesii*	–	–	66.93	Botanical
*Rhus longipes*	–	5.84	–	Botanical
*Ricinodendron rautanenii*	2.95	1.55	–	Botanical
*Stantwasokwe*	–	5.32	–	Tonga
*Sterculia quinqueloba*	12.03	–	–	Botanical
*Strychnos innocua*	–	2.79	–	Botanical
*Terminalia sericea*	–	2.05	4.23	Botanical
*Uvariastrum hexaloboides*	1.71	–	–	Botanical
*Vangueriopsis lanciflora*	2.14	–	–	Botanical
*Ximenia americana*	–	2.59	–	Botanical
*Zanha africana*	10.75	8.42	–	Botanical
*?1 (Not identified)*	–	1.33	–	Not identified

**Table 5:** Species importance value indices of large trees (≥ 5 cm DBH) per site. (Note: A dash means that a species was not found at the site.).

**Species**	**Kabompo**	**Namwala**	**Sesheke**	**Language of the species name**
*Acacia ataxacantha*	–	–	–	Botanical
*Acacia erioloba*	–	–	5.30	Botanical
*Afzelia quanzensis*	1.97	1.00	–	Botanical
*Albizia versicolor*	0.89	1.00	–	Botanical
*Amblygonocarpus andongensis*	4.17	–	–	Botanical
*Baikiaea plurijuga*	48.39	163	149.33	Botanical
*Baphia massaiensis*	33.09	16.00	–	Botanical
*Bauhinia petersiana*	–	–	2.30	Botanical
*Brachystegia boehmii*	–	–	–	Botanical
*Brachystegia longifolia*	8.31	–	–	Botanical
*Brachystegia speciformis*	18.98	–	–	Botanical
*Burkea africana*	8.98	–	2.00	Botanical
*Cassia abbreviata*	–	6.19	–	Botanical
*Combretum celastroides*	–	13.00	–	Botanical
*Combretum hereroense*	–	–	30.99	Botanical
*Combretum imberbe*	–	1.07	–	Botanical
*Combretum molle*	–	6.54	–	Botanical
*Commiphora mollis*	–	7.63	–	Botanical
*Dialium engleranum*	2.94	–	–	Botanical
*Dichrostachys cinerea*	–	–	2.55	Botanical
*Diospyros batocana*	3.99	–	–	Botanical
*Diplorhynchus candylocarpon*	14.09	27.00	8.00	Botanical
*Erythrophleum africanum*	–	–	10.95	Botanical
*Eucalyptus (Exotic species)*	–	–	–	Botanical
*Ficus sycomorus*	–	–	8.14	Botanical
*Guibourtia coleosperma*	3.00	2.00	–	Botanical
*Hymenocardia acida*	0.63	2.00	–	Botanical
*Khaya nyasica*	–	1.65	–	Botanical
*Lannea discolor*	–	–	–	Botanical
*Lannea stuhlmannii*	–	6.26	–	Botanical
*Leza*	–	0.91	–	Tonga
*Lonchocarpus nelsii*	–	–	18.29	Botanical
*Magwilinti*	–	1.99	–	Chewa
*Markhamia obtusifolia*	6.98	–	–	Botanical
*Markhamia obtusifolia*	–	4.36	–	Botanical
*Markhamia zanzibarica*	–	–	–	Botanical
*Matu*	–	2.21	–	Tonga
*Mubangabanga*	–	2.22	–	Tonga
*Mubeba*	1.72	–	–	Luvale
*Muhaswa*	4.46	–	–	Luvale
*Muhuhu*	24.65	–	–	Luvale
*Mukamba*	–	4.19	–	Tonga
*Mukenge*	12.80	–	–	Luvale
*Mukube*	2.12	–	–	Luvale
*Muleyambezo*	–	3.04	–	Tonga
*Mumbukushu*	1.05	–	–	Luvale
*Musenene*	0.70	–	–	Luvale
*Musungwa*	0.83	–	–	Luvale
*Nankhala*	–	–	–	Tonga
*Ochna pulchra*	–	–	2.08	Botanical
*Pseudolachnostylis maprouneifolia*	19.75	3.00	5.00	Botanical
*Pteleopsis anisoptera*	35.45	–	–	Botanical
*Pterocarpus angolensis*	4.42	22.00	5.00	Botanical
*Pterocarpus antunesii*	–	–	25.34	Botanical
*Ricinodendron rautanenii*	21.20	2.00	4.00	Botanical
*Sclerocarya caffra*	–	–	–	Botanical
*Securidaca longepedunculata*	0.62	–	–	Botanical
*Sterculia quinqueloba*	1.92	–	–	Botanical
*Strophanthus welwitschii*	–	–	6.71	Botanical
*Strychnos potatorum*	–	6.16	–	Botanical
*Strychnos pungens*	0.70	–	–	Botanical
*Terminalia sericea*	–	–	8.42	Botanical
*Uvariastrum hexaloboides*	4.29	–	–	Botanical
*Vangueriopsis lanciflora*	0.78	–	–	Botanical
*Ximenia americana*	–	–	–	Botanical
*Zanha africana*	5.45	13.00	–	Botanical
*?1 (Not identified)*	0.66	–	–	Not identified
*?2 (Not identified)*	–	2.73	–	Not identified
*?3 (Not identified)*	–	7.65	–	Not identified
*?4 (Not identified)*	–	–	5.34	Not identified

### 1.3 Carbon stock per species per site

See Table 6 and Supplementary information 1 and 2.

**Table 6:** Carbon stock (t C ha^−1^) per species per site.

**Site**	**Species name**	**Above-ground carbon stock of standing trees (dead and live)**	**Below-ground carbon stock of standing trees (dead and live) trees**	**Number of trees recorded (%)**	**Language of the species name**
Kabompo	*Afzelia quanzensis*	0.031	0.011	0.29	Botanical
Kabompo	*Albizia verscolor*	0.075	0.025	0.07	Botanical
Kabompo	*Amblygonocarpus andongensis*	0.485	0.161	0.72	Botanical
Kabompo	*Baikiaea plurijuga*	7.928	2.637	10.96	Botanical
Kabompo	*Baphia massaiensis*	0.923	0.344	21.27	Botanical
Kabompo	*Brachystegia longifolia*	0.865	0.291	1.59	Botanical
Kabompo	*Brachystegia speciformis*	2.861	0.940	4.33	Botanical
Kabompo	*Burkea africana*	0.751	0.254	1.59	Botanical
Kabompo	*Dialium engleranum*	0.203	0.072	0.94	Botanical
Kabompo	*Diospyros batocana*	0.185	0.066	1.37	Botanical
Kabompo	*Diplorhynchus candylocarpon*	0.195	0.075	6.85	Botanical
Kabompo	*Friesodielsia obovata*	0.001	0.000	0.14	Botanical
Kabompo	*Guibourtia coleosperma*	0.094	0.033	0.43	Botanical
Kabompo	*Hymenocardia acida*	0.003	0.001	0.07	Botanical
Kabompo	*Kabompo1? (Not identified)*	0.010	0.004	0.07	Not identified
Kabompo	*Kapasa ka lyongono*	0.000	0.000	0.07	Luvale
Kabompo	*Markhamia obtusifolia*	0.069	0.026	1.73	Botanical
Kabompo	*Mubangabanga*	0.001	0.000	0.36	Luvale
Kabompo	*Mubeba*	0.052	0.019	0.43	Luvale
Kabompo	*Muhaswa*	0.062	0.023	0.87	Luvale
Kabompo	*Muhoho*	0.000	0.000	0.07	Luvale
Kabompo	*Muhuhu*	1.832	0.641	9.08	Luvale
Kabompo	*Mukenge*	0.558	0.201	4.83	Lunda
Kabompo	*Mukube*	0.019	0.007	0.43	Luvale
Kabompo	*Mumbukushu*	0.013	0.005	0.65	Luvale
Kabompo	*Musenene*	0.018	0.007	0.07	Luvale
Kabompo	*Musungwa*	0.032	0.011	0.14	Luvale
Kabompo	*Pseudolachnostylis maprouneifolia*	1.783	0.618	5.62	Botanical
Kabompo	*Pteleopsis anisoptera*	1.959	0.697	15.79	Botanical
Kabompo	*Pterocarpus angolensis*	0.166	0.057	0.50	Botanical
Kabompo	*Ricinodendron rautanenii*	2.654	0.893	5.55	Botanical
Kabompo	*Securidaca longepedunculata*	0.001	0.001	0.07	Botanical
Kabompo	*Sterculia quinqueloba*	0.024	0.009	0.58	Botanical
Kabompo	*Strychnos pungens*	0.003	0.001	0.14	Botanical
Kabompo	*Uvariastrum hexaloboides*	0.029	0.011	0.94	Botanical
Kabompo	*Vangueriopsis lanciflora*	0.003	0.001	0.29	Botanical
Kabompo	*Zanha africana*	0.038	0.014	1.08	Botanical
Namwala	*Afzelia quanzensis*	0.000	0.000	0.07	Botanical
Namwala	*Albizia verscolor*	0.019	0.007	0.07	Botanical
Namwala	*Baikiaea plurijuga*	12.835	4.421	32.60	Botanical
Namwala	*Baphia massaiensis*	0.203	0.074	13.55	Botanical
Namwala	*Cassia abbreviata*	0.055	0.020	0.86	Botanical
Namwala	*Combretum celastroides*	0.236	0.084	6.77	Botanical
Namwala	*Combretum imberbe*	0.007	0.003	0.13	Botanical
Namwala	*Combretum molle*	0.108	0.039	1.99	Botanical
Namwala	*Combretum zeyheri*	0.000	0.000	0.07	Botanical
Namwala	*Commiphora mollis*	0.149	0.053	1.20	Botanical
Namwala	*Dichrostachys cinerea*	0.000	0.000	0.07	Botanical
Namwala	*Diplorhynchus candylocarpon*	0.389	0.147	11.29	Botanical
Namwala	*Friesodielsia obovata*	0.032	0.011	9.16	Botanical
Namwala	*Guibourtia coleosperma*	0.067	0.024	0.40	Botanical
Namwala	*Hymenocardia acida*	0.018	0.007	0.33	Botanical
Namwala	*Ibu*	0.000	0.000	0.07	Ila
Namwala	*Lannea stuhlmannii*	0.036	0.013	0.73	Botanical
Namwala	*Leza*	0.001	0.001	0.07	Tonga
Namwala	*Mang'omba*	0.001	0.000	0.07	Tonga
Namwala	*Markhamia obtusifolia*	0.023	0.009	2.32	Botanical
Namwala	*Matu*	0.022	0.008	0.40	Tonga
Namwala	*Moonze*	0.000	0.000	0.07	Tonga
Namwala	*Mubangabanga*	0.059	0.020	0.13	Tonga
Namwala	*Mugwirinti*	0.009	0.003	0.20	Tonga
Namwala	*Mukamba*	0.011	0.004	0.40	Tonga
Namwala	*Muleyambezo*	0.038	0.013	0.33	Tonga
Namwala	*Mung'omba*	0.000	0.000	0.07	Tonga
Namwala	*Mutungambabala*	0.000	0.000	0.13	Tonga
Namwala	*Mutwamaila*	0.000	0.000	0.07	Tonga
Namwala	*Mwingili*	0.000	0.000	0.07	Tonga
Namwala	*Namwala1? (Not identified)*	0.139	0.046	0.20	Not identified
Namwala	*Namwala2?(Not identified)*	0.009	0.003	0.13	Not identified
Namwala	*Pericopsis angolensis*	0.065	0.023	0.20	Botanical
Namwala	*Pseudolachnostylis maprouneifolia*	0.030	0.011	0.73	Botanical
Namwala	*Pterocarpus angolensis*	0.429	0.155	9.63	Botanical
Namwala	*Rhus longipes*	0.002	0.001	0.53	Botanical
Namwala	*Ricinodendron rautanenii*	0.040	0.014	0.27	Botanical
Namwala	*Stantwasokwe*	0.003	0.001	0.86	Tonga
Namwala	*Strychnos innocua*	0.002	0.001	0.13	Botanical
Namwala	*Strychnos potatorum*	0.195	0.067	0.93	Botanical
Namwala	*Terminalia sericea*	0.001	0.000	0.13	Botanical
Namwala	*Ximenia americana*	0.001	0.000	0.27	Botanical
Namwala	*Zanha africana*	0.205	0.074	2.32	Botanical
Sesheke	*Acacia ataxacantha*	0.001	0.000	0.75	Botanical
Sesheke	*Acacia erioloba*	0.074	0.026	0.60	Botanical
Sesheke	*Baikiaea plurijuga*	8.200	2.784	35.04	Botanical
Sesheke	*Baphia massaiensis*	0.005	0.002	3.16	Botanical
Sesheke	*Bauhinia petersiana*	0.002	0.001	0.30	Botanical
Sesheke	*Burkea africana*	0.046	0.016	0.15	Botanical
Sesheke	*Combretum hereroense*	0.510	0.182	10.38	Botanical
Sesheke	*Dichrostachys cinerea*	0.005	0.002	0.45	Botanical
Sesheke	*Diplorhynchus candylocarpon*	0.039	0.015	2.86	Botanical
Sesheke	*Erythrophleum africanum*	0.316	0.112	2.71	Botanical
Sesheke	*Ficus sycomorus*	0.168	0.058	0.75	Botanical
Sesheke	*Friesodielsia obovata*	0.029	0.010	7.07	Botanical
Sesheke	*Hippocratea africana*	0.001	0.000	0.30	Botanical
Sesheke	*Lonchocarpus nelsii*	0.114	0.042	4.21	Botanical
Sesheke	*Markhamia zanzibarica*	0.007	0.002	1.05	Botanical
Sesheke	*Namulomo*	0.010	0.003	2.71	Lozi
Sesheke	*Ochna pulchra*	0.003	0.001	0.15	Botanical
Sesheke	*Pseudolachnostylis maprouneifolia*	0.048	0.017	6.47	Botanical
Sesheke	*Pterocarpus angolensis*	0.050	0.018	0.60	Botanical
Sesheke	*Pterocarpus antunesii*	0.303	0.112	15.34	Botanical
Sesheke	*Ricinodendron rautanenii*	0.076	0.027	0.75	Botanical
Sesheke	*Sesheke1?(Not identified)*	0.031	0.012	0.90	Not identified
Sesheke	*Strophanthus welwitschii*	0.016	0.006	0.45	Botanical
Sesheke	*Terminalia sericea*	0.060	0.023	2.86	Botanical

## 2 Experimental design, materials and methods

Our sampling strategy and methods are fully described in Ngoma et al. [1] and its cited references. This section only presents the pictorial processes that we followed to collect our samples to develop below-ground (Section 2.1) and above-ground biomass (Section 2.2) models.

### 2.1 Sample collection process for developing below-ground biomass models

**Fig. 1:**
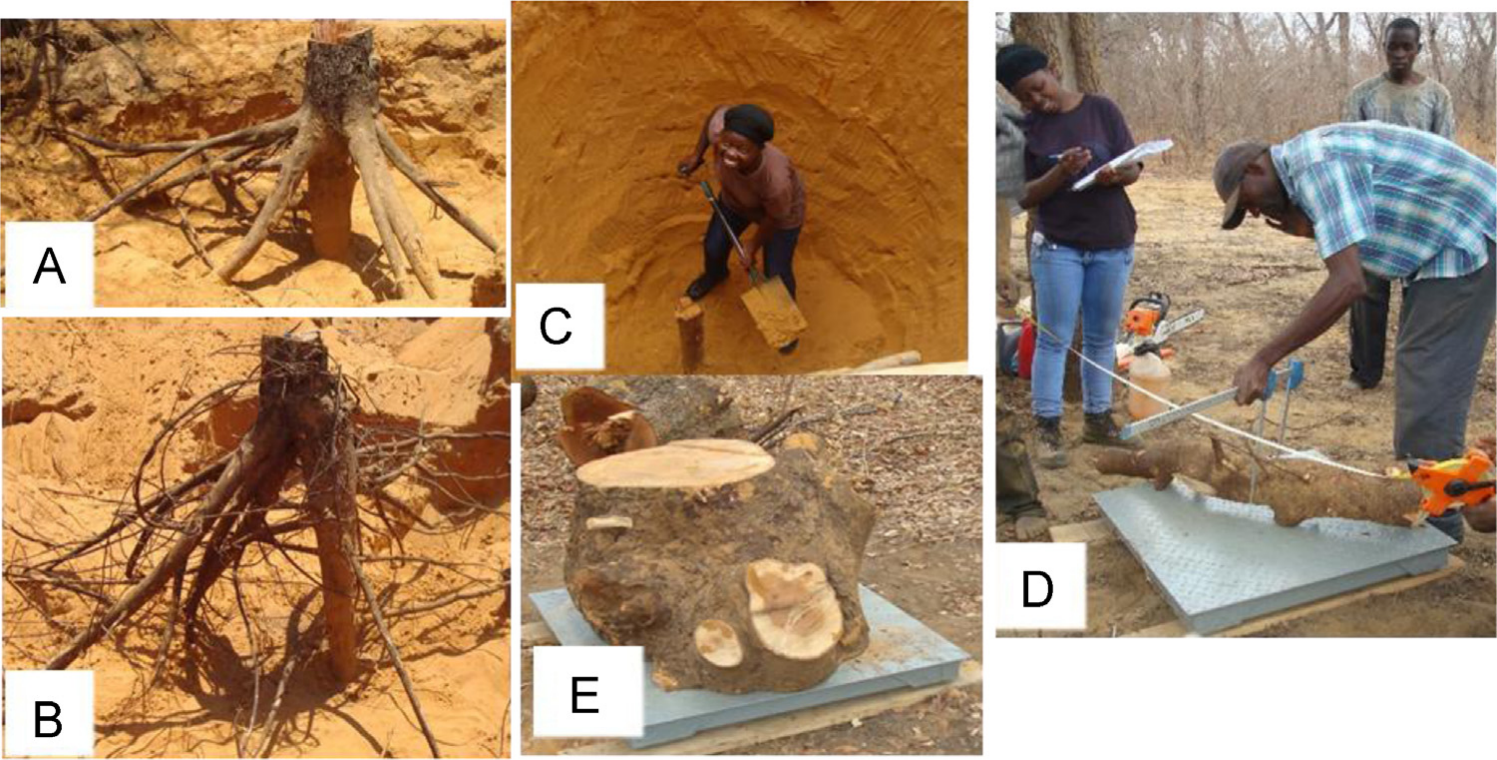
Below-ground sample collection process. Exposed roots are shown in (A) and (B), a taproot is followed in (C), the root's mid-diameter and length are measured in (D) and the root is weighed in (E).

Before felling a tree, we first measured total tree height, bole height, DBH, and crown diameters. The uprooting process started by first exposing all roots connecting directly to the taproot (Fig. 1A and B). We followed both lateral and taproots till they tapered to ≤ 5 mm in diameter (Fig. 1C). We recorded rooting distance and depth for each recorded root. Big root mid-diameters (≥ 5 cm diameter) and their lengths were also measured (Fig. 1D). All roots were weighed immediately after excavation to get their green weight (Fig. 1E).

### 2.2 Sample collection process for developing above-ground biomass models

**Fig. 2:**
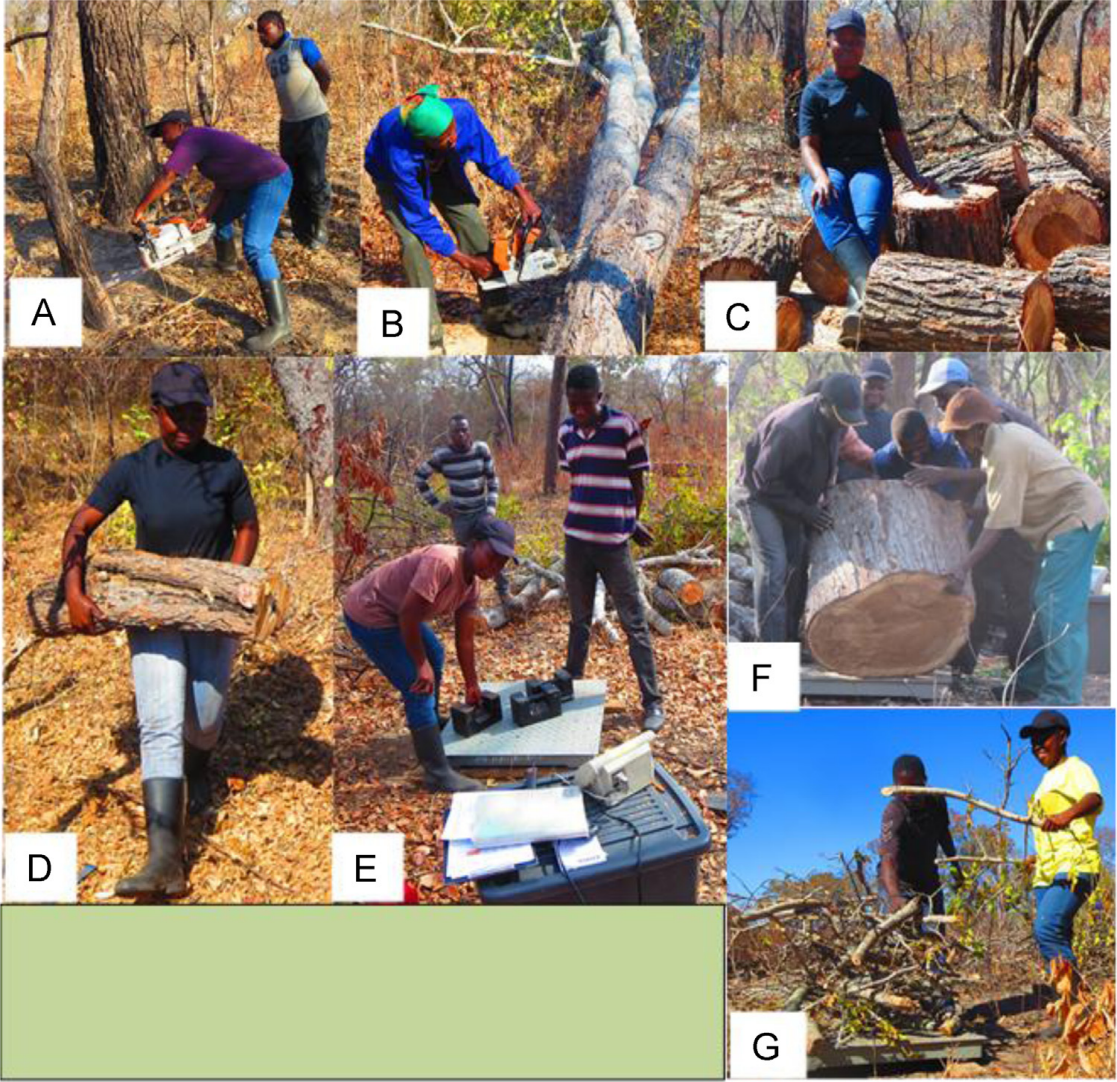
Above-ground sample collection process. Trees are felled (A), cross-cuts (B), and billets (C) are prepared and taken for weighing (D) but, first, the scale is calibrated (E). Large billets (F) and small billets (< 10 cm mid-diameter) including twigs and leaves (G) are weighed.

The felled tree was then cross cut (Fig. 2B) into small billets (Fig. 2C) to unable lifting (Fig. 2D) of the pieces for weighing. However, before weighing, the scale had to be calibrated (Fig. 2E). Large pieces (≥ 10 cm mid diameter) were weighed individually (Fig. 2F) while small pieces (< 10 cm mid-diameter) were weighed as batches together with their twigs and leaves (Fig. 2G).

## Appendix A Supplementary material

Supplementary Information 1: Parameters of all surveyed small trees (< 5 cm DBH) (See excel file available in electronic format).

.

Supplementary Information 2: Parameters of all surveyed large trees (≥ 5 cm DBH) (See excel file available in electronic format).
