# Supplementary material for: Data for developing allometric models and evaluating carbon stocks of the Zambezi Teak Forests in Zambia
Source: Data Brief. 2018 Feb 28;17:1361–73. doi: 10.1016/j.dib.2018.02.057 (PMC5854870; doi:10.1016/j.dib.2018.02.057)
Supplement: Supplementary file 1 — Transparency document. [file mmc1.docx]

**CONFLICT OF INTEREST**

All authors have seen and approved the final version of the manuscript being submitted, and declare that no conflict of interest exists.
